# Kidney and vascular involvement in Alagille syndrome

**DOI:** 10.1007/s00467-024-06562-8

**Published:** 2024-10-24

**Authors:** Bruno Ranchin, Marie-Noelle Meaux, Malo Freppel, Mathias Ruiz, Aurelie De Mul

**Affiliations:** 1 Centre de Référence des Maladies Rénales Rares, Hôpital Femme Mère Enfant, Hospices Civils de Lyon, Bron, France; 2 Faculté de Médecine Lyon Est, Université de Lyon, Lyon, France; 3 INSERM, UMR 1033, Faculté de Médecine Lyon Est, Université de Lyon, Lyon, France; 4 Service d’Hépato-gastroentérologie pédiatrique, Centre de Référence de l’atrésie des voies biliaires et des cholestases génétiques, Hôpital Femme Mère Enfant, Hospices Civils de Lyon, Bron, France

**Keywords:** Alagille syndrome, Kidney, Vascular, Congenital anomalies of the kidney and urinary tract, Renal artery stenosis

## Abstract

**Abstract:**

Alagille syndrome (ALGS) is an autosomal dominant, multisystemic disease with a high interindividual variability. The two causative genes *JAG1* and *NOTCH2* are expressed during kidney development, can be reactivated during adulthood kidney disease, and Notch signalling is essential for vascular morphogenesis and remodelling in mice. Liver disease is the most frequent and severe involvement; neonatal cholestasis occurs in 85% of cases, pruritus in 74%, xanthomas in 24% of cases, and the cumulative incidences of portal hypertension and liver transplantation are 66% and 50% respectively at 18 years of age. Stenosis/hypoplasia of the branch pulmonary arteries is the most frequent vascular abnormality reported in ALGS. Kidney involvement is present in 38% of patients, and can reveal the disease. Congenital anomalies of the kidney and urinary tract is reported in 22% of patients, hyperchloremic acidosis in 9%, and glomerulopathy and/or proteinuria in 6%. A decreased glomerular filtration rate is reported in 10% of patients and is more frequent after liver transplantation for ALGS than for biliary atresia. Kidney failure has been frequently reported in childhood and adulthood. Renal artery stenosis and mid aortic syndrome have also frequently been reported, often associated with hypertension and stenosis and/or aneurysm of other large arteries. ALGS patients require kidney assessment at diagnosis, long-term monitoring of kidney function and early detection of vascular complications, notably if they have undergone liver transplantation, to prevent progression of chronic kidney disease and vascular complications, which account for 15% of deaths at a median age of 2.2 years in the most recent series.

**Graphical abstract:**

**Figure:**
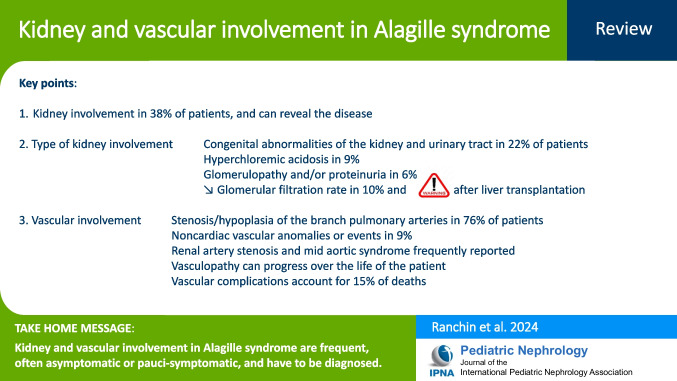
A higher resolution version of the Graphical abstract is available as Supplementary information

**Supplementary Information:**

The online version contains supplementary material available at 10.1007/s00467-024-06562-8.

## Introduction

Alagille syndrome (ALGS) is an autosomal dominant, multisystemic disease, with a high interindividual variability [1, 2] and a frequency that is estimated to be between one in 30,000 to 50,000 live births [3]. Two causative genes have been identified, *JAG1* and *NOTCH2*; these encode two proteins of the Notch signalling pathway: the ligand Jagged1 and the receptor Notch 2. Jagged1 and Notch 2 are transmembrane proteins whose interaction causes cleavage and nuclear translocation of an intracellular fragment of Notch 2, which interacts with transcription factors to influence gene expression [4, 5]. A pathogenic *JAG1* variant or deletion is observed in 94.3% of patients, and a *NOTCH2* variant in 2.5% [6].

**Fig. 1:**
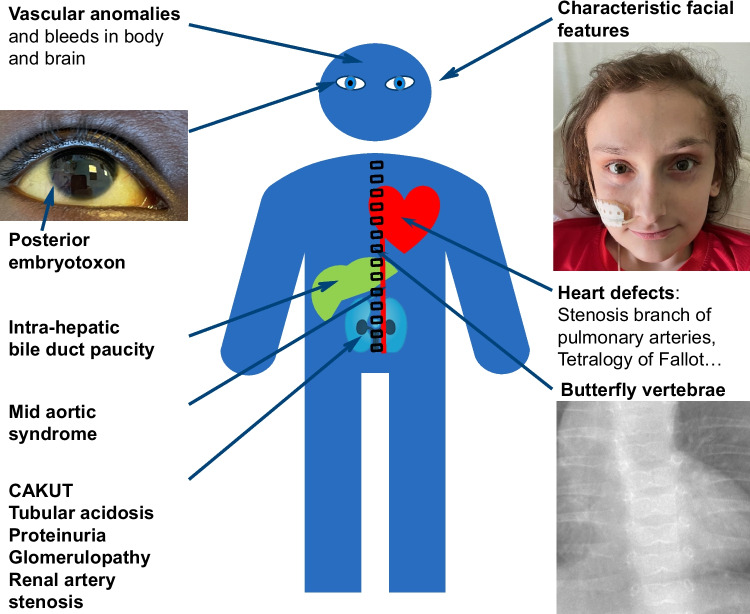
Clinical features of Alagille syndrome. Written consent for publication of these images was obtained from the patients and their parents

ALGS was first described as the association of intrahepatic bile duct paucity with at least three of the following major clinical features: chronic cholestasis, cardiac disease, skeletal anomalies, ocular anomalies, and characteristic facies [2]. Since this initial description, patients with mild or subclinical liver involvement and additional disease features, such as kidney or vascular involvement, have been described [7–9] (Fig. 1).

Liver disease is the most frequent and severe involvement in ALGS. It is reported in 95% of patients; 85% have neonatal cholestasis, 74% pruritus, and 24% xanthomas [1]. By 18 years of age, 69% of patients developed portal hypertension in the largest series published to date, including 1433 children [1]. The cumulative incidence of liver transplantation at 5, 10 and 18 years was, in this series, 27.1% (95% CI, 24.3–30.1), 37.8% (95% CI, 34.2–41.3) and 50.4% (95% CI, 45.4–55.2), respectively. The primary indications for liver transplantation (LT) were complications of cholestasis (refractory pruritus, growth retardation, xanthomas, metabolic bone disease, and/or fat-soluble vitamin deficiency) in 72% of cases and portal hypertension in 30% [1]. Native liver survival (defined as patient survival and absence of LT) at 5, 10 and 18 years was 66.8%, 54.4% and 40.3%, respectively; furthermore, a median total bilirubin level ≥ 5 mg/dl between 6 and 12 months was associated with poorer native liver survival [1].

Cardiac involvement is also extremely frequent; it is diagnosed in 91% of patients [1], the most common abnormality being stenosis/hypoplasia of the branch pulmonary arteries followed by the tetralogy of Fallot [10]. Characteristic facies are reported to be present in 91% of patients [1]: typically triangular, with prominent forehead, deeply set eyes, moderate hypertelorism, pointed chin, and bulbous tip of the nose [11]. Posterior embryotoxon is present in 51%, and butterfly vertebrae in 44% of patients [1].

## Kidney involvement

### Pathophysiology

*NOTCH2* and *JAG1* are expressed during kidney development and are required for the formation of tubules and glomeruli during nephrogenesis [8, 12–14]. Notch2-deficient mice have multiple congenital kidney cysts arising mainly from the proximal tubule [15]. Notch signalling is also involved in regulating the glomerular filtration barrier, and abnormal activation of Notch 1 signalling in the glomerular endothelium inhibits the expression of VE-cadherin, which induces albuminuria through the transcription factors Snai1 and Erg [16]. The Notch pathway could be reactivated in adult-onset kidney diseases [8].

Cholestasis is also associated with kidney dysfunction and secondary lesions; this cholemic nephropathy is poorly described and its pathophysiology only partially elucidated [17]. It has recently been reported, using a murine model of obstructive cholestasis, that cholemic nephropathy is due to biliary acids, and that the renal apical sodium dependent bile acid transporter of proximal tubular cells has a main role in the kidney toxicity of biliary acids [18].

### Frequency

When reported, a kidney involvement is present in 38% of patients (Table 1) [1, 19–26] and this has led some experts to consider kidney anomalies as a disease-defining feature [8, 25]. Kidney involvement is often asymptomatic and can reveal the disease even in adults [27–31]. However, the published series primarily focus on paediatric cases, are cross-sectional, and are typically initiated by paediatric hepatologists. Kidney investigations are only partially described in most cases and exhibit wide variations. However, the landmark series reported by Habib et al. is unique in that all children underwent biopsy and pathology investigation despite exhibiting very mild renal symptoms [19].

**Table 1:** Frequency of kidney involvement

First author (year)	Total population, *n*	Median age, year [range] or (IQR)	Patients with kidney involvement, *n* (%)
Habib (1987) [19]	80	children	20 (25)
Hofenberg (1995) [20]	26	nr [1-31]	5 (19)
Emerick (1999) [21]	69	nr	28 (41)
Quiros-Tejeira (1999) [22]	30	8.7 [0.6–28.9]	15 (50)
Wang (2008) [23]	6	3.5 [1.6–4.1]	2 (33)
Subramaniam (2011) [24]	117	children	27 (23)
Kamath (2012) [25]	187	nr	73 (39)
Di Pinto (2018) [26]	21	12.3 [2.6–19.2]	18 (86)
Vandriel (2023) [1]	1275	6.0 (2.5–12.2)	500 (39)
Total	1811		688 (38)

*IQR* interquartile rang, *nr* not reported

### Type of kidney involvement

The frequency of different types of kidney involvement is reported in Table 2 [19–26].

**Table 2:** Type of kidney involvement

First author (year)	Patients		CAKUT				Hyper-chloremic acidosis, *n* (%)	Glomerulopathy or proteinuria, *n* (%)
	*n*	With kidney involvement, *n* (%)	Hypoplasia dysplasia, *n* (%)	Cysts, *n* (%)	Agenesia, *n* (%)	Uropathy and/or lithiasis, *n* (%)		
Habib (1987) [19]	80	20 (25)	1 (1)	0	0	0	10 (12)	18 (22)
Hoffenberg (1995) [20]	26	5 (19)	1 (4)	1 (4)	1 (4)	0	2 (8)	0
Emerick (1999) [21]	69	28 (40)	14 (20)	5 (7)	0	5 (7)	10 (14)	0
Quiros-Tejeira (1999) [22]	30	15 (50)	8 (27)	3 (10)	0	0	0	0
Wang (2008) [23]	6	2 (33)	2 (33)	0	0	0	0	0
Subramaniam (2011) [24]	117	27 (23)	9 (8)	nr	1 (1)	3 (3)	16 (14)	0
Kamath (2012) [25]	187	73 (39)	43 (23)	nr	nr	14 (7)	7 (4)	4 (2)
Di Pinto (2018) [26]	21	18 (85)	6 (29)	0	1 (5)	0	2 (10)	11 (52)
Total	536	188 (35)	117 (22)				47 (9)	33 (6)

*N* number of, *nr* not reported

### Congenital anomalies of the kidney and urinary tract (CAKUT)

The most frequent type of kidney involvement is CAKUT, which is reported in 22% of patients (Table 2). Many isolated cases have been reported [20, 22, 27, 32–35], with cortical cysts [22, 36–38] or multicystic dysplastic kidney [37] in some patients. CAKUT can be diagnosed in children or adult patients with known ALGS, but the syndrome can also be revealed by symptomatology caused by kidney hypo-dysplasia and/or hypertension in children [28] and adult patients [27, 29–31, 39].

### Glomerulopathy and pathology reports

The frequency of glomerulopathy/proteinuria is highly variable (Table 2) [19–26], and this is probably in line with the type of investigations carried out. The pathophysiology of glomerular lesions is multifactorial, secondary: to nephron reduction caused by hypo-dysplasia, to lipid deposits secondary to high hyperlipaemia [19], and/or to hyperfiltration due to the high serum biliary acids [18].

Among 80 children affected by ALGS, Habib et al. reported on kidney pathology studies in 26 children with ALGS and no kidney dysfunction in 12, minor anomalies (metabolic acidosis under cholestyramine therapy, mild proteinuria and/or mildly decreased glomerular filtration rate, GFR) in 13, and a severe kidney dysfunction due to bilateral hypoplasia in one child [19]. The authors describe that in 18 of these patients, at a median age of 1.3 [range: 0.25–17] years, glomerular lesions were characterized by a mesangiolipidosis. The mesangium had a fibrillar appearance on light microscopy (LM), with widespread lipid vacuoles in the mesangial matrix on electron microscopy (EM). In 10 patients, this aspect was associated with the presence of mesangial foam cells.

Russo et al. compared kidney pathology at autopsy of four children with ALGS and five children with other cholestatic diseases [40]. The four with ALGS died within nine days of LT, and among these the two older children (6.5 and 7.0 years of age) presented heavy proteinuria at the time of admission for LT. EM found in all nine cases focal areas of thickening and vacuolisation with electron-dense particles in both glomerular basement membrane and mesangium. LM found in the two proteinuric ALGS patients an aspect of membranous nephropathy with swollen glomeruli, widened mesangial matrix, and thickened double contoured capillary walls with argyrophilic spikes due to the accumulation of lipids in the glomerular membranes. Immunofluorescence, used in one of these two patients, found immunoglobin and complement deposits on glomerular capillary loops. The two proteinuric ALGS patients also had severe tubulointerstitial changes with thickening of tubular basement membranes, accumulation of lipids in these membranes, tubular atrophy and chronic interstitial inflammation. On EM the glomerular and tubular basement membranes of these two patients were irregularly thickened with vacuoles containing osmiophilic dense particles. All four ALGS patients presented interstitial chronic nephritis and tubular atrophy, which was associated with cysts in two of them.

There are also some isolated reports. Chronic glomerular lesions with focal and segmental glomerulosclerosis (FSGS) as well as chronic tubular lesions with thickening of basement membranes and subcortical cysts were found in a 20-day-old child with kidney failure (KF) [41]. Severe glomerulosclerosis, interstitial fibrosis and tubular atrophia, without signs of mesangiolipidosis, were reported in a 46-year-old male presenting with KF and bilateral small kidney [30]. A five-year-old girl was biopsied because of persistent isolated proteinuria without renal insufficiency or haematuria; glomerular basement membrane irregularities, mesangial sclerosis, and FSGS were found using LM, and a few lipid inclusions within the mesangium but extensive inclusions along the glomerular basement membrane were found using EM [38]. Basement membrane and mesangial lipidosis, cholemic nephropathy, and kidney dysplasia were described in a 19-year-old male with KF [42].

Kidney pathology investigations were performed in 4/92 ALGS patients described by Emerick et al., showing renal lipidosis in a child with proteinuria; cystic tubular dilation in an infant with renal insufficiency and tubular acidosis; glomeruli filled with foamy macrophages, dilated tubules, and sclerotic glomeruli in one other infant who did not have clinical kidney disease and dilated tubules with cystic spaces; and lipidosis in one infant who had infantile renal insufficiency and renal tubular acidosis [21]. A kidney biopsy was performed in 4/73 children with ALGS and kidney involvement who were reported by Kamath et al.; renal lipidosis was found in two children, FSGS in one, and ciclosporin toxicity and IgA nephropathy in another [25].

### Kidney failure

A decreased GFR seems frequent in ALGS; when reported it was found in 10% of patients and KF in 2% (Table 3) [19–23, 25, 26]. Numerous isolated cases have also been published [2, 27–32, 34, 35, 41–44]. KF has been reported in childhood as soon as 20 days of age [41]. Decreased GFR before LT, as well as one and two years post-LT was more frequent in 91 ALGS patients than in 236 age-matched patients with biliary atresia (18, 22 and 17% versus 5, 8 and 7%, respectively) [45].

**Table 3:** Decreased glomerular filtration rate and kidney failure in Alagille syndrome

First author (year)	Patients, *n*	With kidney involvement, *n* (%)	Median age, years [range]	Decreased glomerular filtration rate, *n* (%)	Kidney failure, *n* (%)
Habib (1987) [19]	80	20 (25)	children	6 (7)	1 (1)
Hoffenberg (1995) [20]	26	5 (19)	nr [1-31]	0	0
Emerick (1999) [21]	69	28 (40)	nr	1 (1)	0
Quiros-Tejeira (1999) [22]	30	15 (50)	8.7 [0.6–28.9]	6 (20)	4 (13)
Wang (2008) [23]	6	2 (33)	3.5 [1.6–4.1]	0	0
Kamath (2012) [25]	187	73 (39)	nr	17 (9)	3 (2)
Di Pinto (2018) [26]	21	18 (85)	12.3 [2.6–19.2]	10 (48)	2 (10)
Total	419	161 (38)		40 (10)	10 (2)

*n* number of, *n* number of, *IQR* interquartile rate, *nr* non reported

### Hyperchloremic acidosis

Hyperchloremic acidosis has been reported in 9% of patients in published series (Table 2) [19–26]. It is poorly described and in older studies can be, at least partially, due to cholestyramine therapy [19]. This has to be corrected to prevent growth retardation and demineralisation [46] that are frequent in ALGS, due to many factors in addition to acidosis such as cholestasis, malnutrition and fat-soluble vitamin deficiency [7]. Following recommendations in chronic kidney disease (CKD) a serum bicarbonate level of at least 22 mmol/L should be targeted [46].

### Hyponatremia

Hyponatremia is not reported in the literature but can be observed in clinical practice, and can be due to tubular dysfunction. In such cases, serum sodium has to be measured by direct potentiometry to eliminate a false hyponatremia secondary to hyperlipemia [47].

## Vasculopathy

Notch signalling is essential for vascular morphogenesis and remodelling in mice [48–50]. Stenosis/hypoplasia of the branch pulmonary arteries is the most frequent vascular abnormality reported in ALGS, present in 76% (152/200) of patients with ALGS and/or *JAG1* mutation [10]. Many other large arteries can be affected, and noncardiac vascular anomalies or events are reported in 9% (25/268) of patients with ALGS described by Kamath et al. [9]. Moreover vasculopathy can progress over the life of the patient [39, 51, 52] and vascular complications account for 15% of deaths at a median age of 2.2 years in the most recent report [1]. Abnormal retinal vascularity with venous tortuosity is increased in patients with ALGS and can be detected by fundus examination, which could be a non-invasive means of detecting vasculopathy in these patients [49].

### Arteries of the head and neck

Cerebral vasculopathy seems frequent, detected on magnetic resonance imaging with angiography in 5/22 asymptomatic patients at a median age of 6.5 [range: 0.75–30] years and is associated with risk of bleeding or ischemic intracranial events [51]. They have been described on internal carotid [27, 34, 39, 51, 53], basilar trunk [34, 51, 53], middle cerebral [51], and vertebral arteries [34].

### Renal arteries and mid aortic syndrome

Renal artery stenosis (RAS) [9, 22, 25, 27, 29, 32, 33, 35, 39, 52, 54–63], and mid aortic syndrome (MAS) [22, 29, 32, 35, 39, 52–55, 60, 61, 63–65] are frequently reported (Table 4). Coarctation of the aorta has been reported in 4/200 patients [10] and 2/43 patients [22] and a MAS in 1/43 patients [22] in two series. However, as stenosis of aorta or renal arteries in ALGS is reported to be frequently asymptomatic or pauci-symptomatic [59], the frequency of lesions reported in the literature may be underestimated. For instance, RAS was diagnosed by angiography or computed tomography (CT) in 2/25 children at a mean age of 6 years [range: 1–17] during pre-LT check-up [58]; furthermore, RAS was found in 6/12 children and young adults after LT on CT at a mean age of 14.7 [range: 1.7–22.9] years, only one of whom was hypertensive [59]. Conversely, these frequencies are likely to be greater than those found in the general population of ALGS patients as these studies included only patients who were to be or had been transplanted. Another point of note is that an association with *JAG1* mutation has been reported in 4/35 families with paediatric MAS, without any other symptoms of ALGS in one patient [64].

**Table 4:** Renal artery stenosis and mid aortic syndrome, published cases

First author (year)	Patients	With renal artery stenosis		With mid aortic syndrome		With arterial hypertension, *n*/total	With other vascular stenosis and/or anevrysms, n/total
	Total, *n*	*n*	Age at diagnosis, years	*n*	Age at diagnosis, years		
Exss (1976) [57]	1	1	10			1/1	0/1
Devloo- Blancquaert (1980) [62]	1	1	0.3			nr	1/1
Labrecque (1982) [32]	1	1	adulthood	1	adulthood	1/1	nr
Kurtz (1987) [61]	1	1	15	1	15	1/1	nr
Shefler (1997) [63]	1	1	16	1	16	1/1	1/1
Bérard (1998) [52]	5	5	3.5, 3.5, 7, 12, 28	2	0.2, 12	5/5	4/5
Quiros-Tejeira (1999) [22]	2	1	childhood	1	childhood	nr	nr
Quek (2000) [65]	1			1	8	0/1	1/1
Raas-Rothschild (2002) [54]	1	1	14	1	14	1/1	1/1
Schlosser (2004) [53]	1			1	30	nr	1/1
Hia (2004) [60]	1	1	13	1	13	0/1	0/1
Kamath (2004) [9]	1	1	nr			1/1	1/1
Hirai (2005) [33]	1	1	10			1/1	0/1
Jacquet (2007) [29]	1	1	30	1	30	1/1	0/1
Yucel (2010) [35]	1	1	38	1	38	1/1	0/1
Kayhan (2010) [56]	1	1	43			nr	1/1
Shrivastava (2010) [27]	2	2	50, 62			2/2	2/2
Kamath (2012) [25]	2	2	nr			nr	nr
Salem (2012) [39]	5*	4	13, 15, 23, 27	5	23, 25, 27, 28	5/5	4/4
Kohaut (2017) [58]	2	2	childhood			nr	1/2
Warejko (2018) [64]	4			4	0, 0.9, 4, 11	4/4	4/4
Sanada (2019) [59]	6	6	1.7, 11.5, 11.8, 15.5, 17.1, 22.9			1/6	5/6
Yokoyama (2020) [55]	1	1	4	1	4	1/1	1/1
Total	42	35	0.3 to 62	22	0 to 38	27/34 (79%)	28/36 (78%)

*nr* non reported, *: One patient previously described at a younger age in Bérard 1998

Arterial hypertension was present at RAS or MAS diagnosis in 79% of published cases (Table 4) and renal dysplasia can also cause hypertension. This hypertension can be present early in life as soon as 3.5 [52], 5 [39], 10 [57], 13 [39], and 14 years of age [39, 54] and can lead to ALGS diagnosis in some patients, as reported in a 27-year-old patient [39]. Blood pressure should be measured regularly in both arms in ALGS patients because this can be asymmetric between the two arms due to a subclavian artery stenosis that has been reported in ALGS [27, 39, 52, 54, 62]. Hypertension is frequently asymptomatic or pauci-symptomatic but increases the risk of vascular complications which were the cause of 15% of the 108 deaths reported in a series of 1433 children at a median age of 2.2 (IQR: 1.5–3.2) years [1].

Stenosis and or aneurysm of other large arteries are also common; these are associated with RAS or MAS in 78% of published cases (Table 4). They have been described in: celiac trunk [39, 52, 53, 56, 58, 59, 63, 64], superior mesenteric [3, 6, 17, 20, 24, 25, 30, 32], hepatic [56, 58, 59, 62, 63], splenic [53, 59], gastroduodenal [56], inferior pancreaticoduodenal [59], coronary [62], iliac [27], and subclavian arteries [27, 39, 52, 54, 62]. Again, these lesions can be asymptomatic and their frequency in AS cohort is probably a function of severity of the disease but also of diagnostic tools used. Thus, stenosis of celiac trunk and hepatic artery seem frequent in ALGS children requiring LT and explored by arteriography or CT: being present in respectively 12 and 10 children among 25 investigated children in one series [58] and 5 and 1 among 12 children or young adults in another series [59].

Intracranial haemorrhage is one of the most frequent causes of death in patients with ALGS. It has been described as a consequence of head trauma, aneurysm, coagulopathy associated with liver disease or spontaneous. Spontaneous intracranial haemorrhage appears to be more common in girls with ALGS [49].

Pathology studies of the arterial wall are scarce; however, myxoid degeneration with ectopic bone formation has been reported in one adult patient with hypoplastic aorta [32], and tight lumen with myo-intimal hyperplasia and slight disruption of the internal limitans on a clinically asymptomatic zone of the left cubital artery in a 28-year-old hypertensive patient [52].

## Proposed screening and follow-up for kidney and vascular manifestations in patients with Alagille syndrome

### Kidney involvement

All children with ALGS should have an ultrasonography of kidneys and urinary tract at diagnosis with measurement of kidney length and investigation for pyelo-ureteral dilatation and/or bladder anomalies. Serum bicarbonate and creatinine should be measured at each blood sampling for liver follow-up, and the urinary proteins over creatinine ratio should be measured at least yearly and more frequently in patients with the most severe forms. Specific attention to kidney function should be given post-liver transplantation.

### Vascular involvement

Blood pressure should be measured at each consultation and annually in both arms, and strictly controlled to reduce the risk of bleeding. Decreased arterial pulsatility and vascular murmurs should be sought in the main arterial pathways.

Cerebrovascular imaging may be considered to detect intracranial vascular anomalies, but timing and modalities are debatable. Retinography could be used for non-invasive screening of vascular anomalies, but this method needs to be validated.

These suggestions are based on our review of the literature, and there are currently no consensus guidelines on this subject.

## Conclusion

Kidney and vascular involvement are frequent in ALGS. They are often asymptomatic or pauci-symptomatic and must be diagnosed in order to prevent CKD progression and kidney sequelae, but also to reduce the risk of vascular complications. Blood pressure should be measured at each consultation and periodically in two arms.

## Supplementary Information

Below is the link to the electronic supplementary material.

Graphical abstract (PPTX 82 KB)
