# Supplementary material for: Kidney and vascular involvement in Alagille syndrome
Source: Pediatr Nephrol. 2024 Oct 24;40(4):891–9. doi: 10.1007/s00467-024-06562-8 (PMC11885393; doi:10.1007/s00467-024-06562-8)
Supplement: Supplementary file 1 — Graphical abstract (PPTX 82 KB) [file 467_2024_6562_MOESM1_ESM.pptx]

## Slide 1
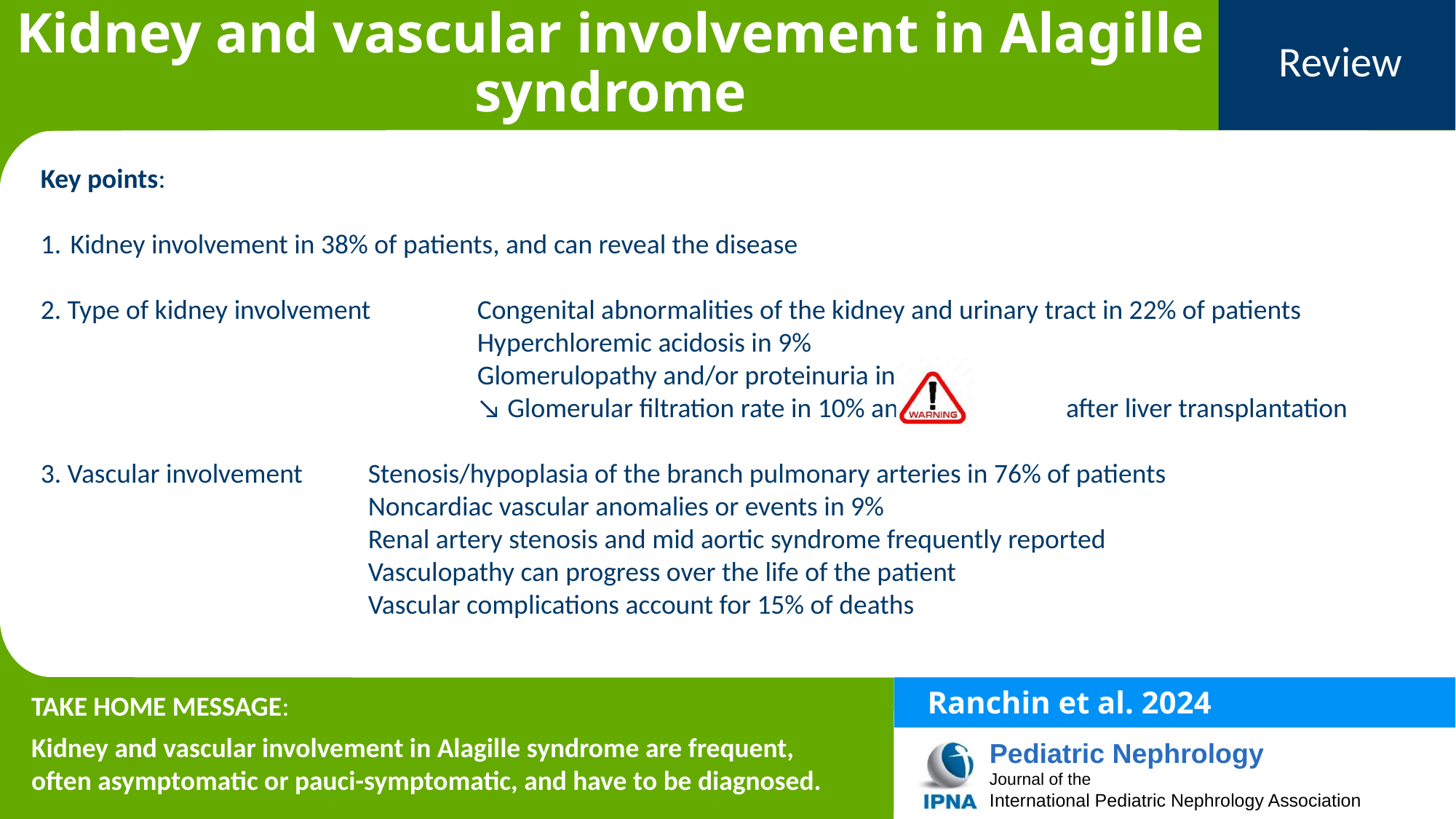

Kidney and vascular involvement in Alagille syndrome
Key points:
Kidney involvement in 38% of patients, and can reveal the disease
2. Type of kidney involvement 	Congenital abnormalities of the kidney and urinary tract in 22% of patients
				Hyperchloremic acidosis in 9%
				Glomerulopathy and/or proteinuria in 6%,
				↘ Glomerular filtration rate in 10% and 	 after liver transplantation
3. Vascular involvement 	Stenosis/hypoplasia of the branch pulmonary arteries in 76% of patients
			Noncardiac vascular anomalies or events in 9%
			Renal artery stenosis and mid aortic syndrome frequently reported
			Vasculopathy can progress over the life of the patient
			Vascular complications account for 15% of deaths
Ranchin et al. 2024
TAKE HOME MESSAGE:
Kidney and vascular involvement in Alagille syndrome are frequent,
often asymptomatic or pauci-symptomatic, and have to be diagnosed.
